# Uncommon and Accessory Electrocardiographic Findings in Brugada Syndrome: A Review

**DOI:** 10.3390/jcm14165895

**Published:** 2025-08-21

**Authors:** Antonino Micari, Paolo Bellocchi, Asya Cautela, Alice Moncada, Matteo Pluchino, Maurizio Cusmà-Piccione, Lilia Oreto, Giampiero Vizzari, Giuseppe Dattilo, Pasquale Crea

**Affiliations:** 1 Cardiology Unit, Department of Clinical and Experimental Medicine, University of Messina, AOU Policlinico “G. Martino”, 98124 Messina, Italy; 2 Pediatric Cardiology Unit, Department of Clinical and Experimental Medicine, University of Messina, AOU Policlinico “G. Martino”, 98124 Messina, Italy; 3 Cardiology Unit, Department of Biomedical and Dental Sciences and Morphological and Functional Imaging, AOU Policlinico “G. Martino”, 98124 Messina, Italy

**Keywords:** Brugada syndrome, electrocardiography, sudden cardiac death, ventricular arrhythmias

## Abstract

Brugada syndrome (BrS) is a cardiac arrhythmic disorder associated with distinctive electrocardiographic (ECG) abnormalities and an increased risk of sudden cardiac death due to ventricular arrhythmias. While the classic BrS ECG pattern is a coved ST-segment elevation in the right precordial leads, a wide spectrum of atypical ECG presentations can mislead the diagnosis. This review discusses rare and under-recognized ECG findings associated with BrS, including its coexistence with right and left bundle branch block, alterations in peripheral leads and in the morphology of the QRS complex, as well as atrioventricular conduction abnormalities. Emphasis is placed on the clinical relevance of these findings, their underlying electrophysiological mechanisms, and their prognostic implications. Recognizing these atypical manifestations is critical to avoid misdiagnosing or failing to recognize the condition in patients with BrS.

## 1. Introduction

Brugada syndrome (BrS) is a hereditary arrhythmic disorder characterized by an abnormal electrocardiogram (ECG) and a predisposition to sudden cardiac death due to ventricular tachyarrhythmias. While the hallmark ECG pattern of BrS is a coved-type ST-segment elevation in the right precordial leads (V1–V2), atypical ECG patterns have been described, complicating the diagnosis. These include the overlap of the Brugada pattern with conduction disturbances such as right or left bundle branch block, the presence of ST-segment abnormalities in inferior and peripheral leads, QRS fragmentation, and atrioventricular conduction disorders.

The clinical importance of recognizing these atypical features cannot be underestimated. Misinterpretation or under-recognition may lead to missed or delayed diagnoses. Furthermore, these uncommon ECG patterns may carry distinct prognostic implications and influence therapeutic strategies. In this review, we explore the spectrum of atypical ECG presentations in BrS, examine the underlying electrophysiological mechanisms, and discuss the implications for diagnosis, risk stratification, and management.

## 2. Coexistence of Right Bundle Branch Block and Brugada Pattern

Although right bundle branch block (RBBB) has classically been considered a benign finding [1,2], it was observed that this conduction abnormality shows a higher prevalence in patients with idiopathic ventricular fibrillation (VF) [3]. Moreover, it is independently associated with a higher cardiovascular risk [4] and an increased risk of all-cause mortality, even in patients without cardiovascular disease [5]. Interestingly, it is a common finding in patients with BrS, as shown by a retrospective study by Maury et al., with a prevalence of 28% [6] in comparison with a prevalence in the general population of 0.2% to 1.3% [5].

The characteristic pattern of BrS may overlap with the QRS alterations of a complete RBBB. Indeed, the R’ wave in the right precordial leads typical of RBBB may conceal the terminal wave of the Brugada pattern. Moreover, complete RBBB shows a depression of the ST segment in the right precordial leads, opposite to the QRS polarity, whereas the Brugada pattern shows a coved ST-segment elevation in these leads.

Therefore, RBBB may mask the Brugada pattern, which should always be ruled out whenever a slight ST-segment elevation is observed in the right precordial leads in the presence of a complete RBBB.

**Figure 1:**
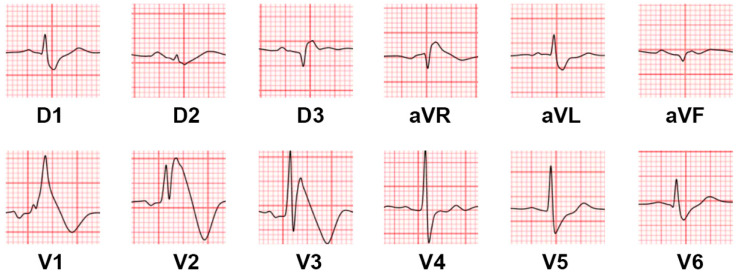
Basal 12-lead surface ECG showing a RBBB with a wide terminal R’ wave in right precordial leads, suggestive of a Brugada type 1 pattern.

As reported by several authors, in such a condition, the Brugada pattern may be unmasked by the transient normalization of the conduction delay [7,8,9]. However, sometimes, terminal QRS widening in the right precordial leads can be so evident as to allow the diagnosis of Brugada syndrome even in the presence of a complete RBBB, as shown in Figure 1.

Another possible solution is a specific electrophysiologic maneuver, named Chiale’s maneuver after the first author who described it. It can unmask the Brugada pattern in cases of complete RBBB through ventricular pacing. It is based on right apical ventricular pacing with appropriately timed atrioventricular intervals, which allow the fusion of the paced QRS, with a left bundle branch block (LBBB) morphology, and the native QRS, with a RBBB morphology. This results in narrow fused QRS complexes in which the Brugada pattern can be eventually observed in the right precordial leads [10].

The pattern can also be unmasked by febrile illness [11] or drugs commonly used to unmask the pattern, such as ajmaline [12]. However, caution should be exercised as the administration of ajmaline in the presence of infrahissian conduction disorders is associated with an increased risk of atrioventricular block [13]. Furthermore, there is evidence that the administration of ajmaline in patients with infrahissian conduction disorders may lead to the development of atypical ventricular tachycardias, such as bundle-to-bundle tachycardia [14].

## 3. Brugada Pattern and Left Bundle Branch Block

Complete LBBB is not a common conduction disturbance in patients with BrS, with a prevalence of 1% [6]. This may be explained by the fact that the diagnosis of BrS occurs at a young age, while left bundle branch block is more common in older populations, with a prevalence that increases steadily from <1% at the age of 50 years to 6% by 80 years of age [15,16].

**Figure 2:**
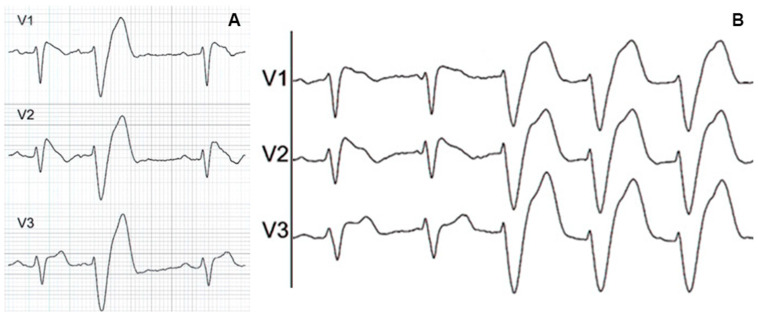
(**A**). Surface ECG showing premature atrial complex conducted with aberration and with a LBBB morphology, masking the Brugada pattern in the precordial leads. (**B**). Right atrial pacing conducted with aberration showing a wide QRS with LBBB morphology, which hides the Brugada pattern normally visible in the precordial leads with narrow QRS. Adapted with permission from Arana-Rueda E. et al. [20].

In complete LBBB, the right ventricle is firstly activated through the right bundle, and then the left ventricle is slowly activated through the septal myocardium. Therefore, electric forces are directed towards the left and posteriorly. This causes a deep S wave to appear in the right precordial leads, leading to the masking of the Brugada pattern, which is hidden within the wide QRS complex. The Brugada pattern may be revealed in cases of intermittent LBBB, as shown in Figure 2.

It has been recently described how Para-Hisian pacing can overcome the LBBB, unmasking an underlying Brugada pattern [17]. Indeed, the ability to narrow the QRS with parahissian pacing would depend on the longitudinal dissociation of the fibers within the His bundle, which would explain that LBBB could be due to a focal proximal interruption of the fibers within the His bundle [18]. Therefore, direct stimulation of the His bundle would allow for a distal capture of these fibers, normalizing the QRS and unmasking a possible Brugada pattern. However, given the very low prevalence of LBBB in patients with Brugada pattern, experience with this maneuver in this population is limited. Furthermore, the need for an invasive approach to perform it limits its applicability in a routine diagnostic workup.

Another diagnostic possibility to unmask the pattern, even in cases of LBBB, is represented by ajmaline tests as reported by Özin et al. [19], always taking into account the risks associated with the use of this drug in patients with infrahissian conduction disorders [13,14].

Arana-Rueda et al. described a newly discovered sodium voltage-gated channel (SCN5A) mutation (p.1449Y>H), which causes both severe sodium-channel dysfunction and complete LBBB, which conceals the Brugada pattern on surface ECG. This demonstrates that this mutation can cause not only the Brugada pattern, but also the development of intraventricular conduction abnormalities [20].

Therefore, the presence of a LBBB in a patient with a suspicion of Brugada syndrome (e.g., in presence of sudden cardiac death or a known SCN5A mutation in the family, in cases of unexplained syncope, especially at night or triggered by fever or certain drugs) highlights the need for electrophysiological evaluation or specific maneuvers to unmask the pattern.

## 4. ST-Segment Depression in Inferior Leads

Although the common presentation of a type 1 Brugada pattern involves conduction abnormalities in right precordial leads, it is not uncommon to observe alterations involving the peripheral leads.

A common but often underdiagnosed sign is the depression of the ST segment in the inferior leads. It is recognizable in approximately 50% of patients with Brugada syndrome, even in those where the pattern in the precordial leads at the fourth intercostal space is not immediately visible [21,22]. In particular, Crea et al. performed an analysis of 87 patients with a spontaneous type 1 Brugada pattern, showing that 47% exhibited ≥0.1 mV ST depression lasting ≥0.08 s in leads II, III, or aVF [21,23]. The pattern can be recognized more easily in inverted mirror image tracing, which can be obtained by flipping the paper and analyzing it with a backlight. Once the ST-segment depression is recognized, the precordial leads can be placed at the second and third intercostal spaces to try to observe the pattern.

It has been described how the electrogenesis of this anomaly may depend on the three-dimensional orientation of the right ventricular outflow tract (RVOT). In particular, a horizontal RVOT is responsible for the genesis of a vector directed superiorly, explaining ST-segment depression in inferior leads, and anteriorly, explaining the Brugada pattern in the precordial leads [24].

ST-segment depression can also be induced by a drug challenge, as Chinushi et al. described in two cases after the administration of pilsicainide [25].

Thus, ST depression in the inferior leads serves as an important, under-recognized ECG sign that may guide a more accurate diagnosis of Brugada syndrome.

A challenge in the differential diagnosis may be represented by ST-segment depression due to myocardial ischemia. However, in this case, the ST segment usually presents a descending or rigid trend, with a possible inversion of the T wave, and it is represented mainly in anterior and lateral leads, while, in patients with Brugada, it is more often an ascending ST-segment depression with normal T waves. Moreover, in case of myocardial ischemia the clinical presentation is highly indicative.

**Figure 3:**
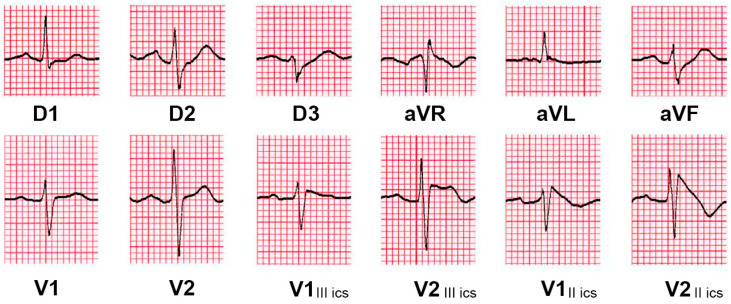
Basal 12-lead surface ECG showing a ST-depression in inferior leads, with an apparently normal ST segment in the right precordial leads at the fourth intercostal space. The precordial leads at the second intercostal space show a type 1 Brugada pattern with a wide terminal R’ wave. The basal ECG allowed us to suspect a Brugada pattern only through the analysis of the inferior leads.

An example of an ECG of a patient with ST-segment depression in the inferior leads, with a normal ST-segment profile at the fourth intercostal space, but showing a type 1 Brugada pattern at the second intercostal space, is shown in Figure 3.

## 5. Other Anomalies of the Peripheral Leads

**Figure 4:**
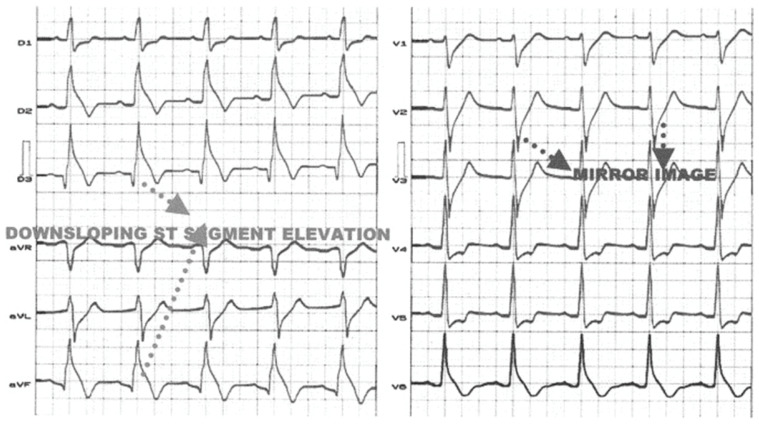
A 12-lead ECG showing a downsloping ST-segment elevation in inferior leads with a prominent J wave. A mirror image can be seen in anterior precordial leads (V2–V3). Reproduced with permission from Riera et al. [30].

While the classic Brugada pattern displays a coved ST-segment elevation in the right precordial leads (V1–V3), studies report that ~9–10% of patients may also show a type 1 ST elevation in at least one peripheral lead, mainly in aVR and in the inferior leads, and this is independently associated with malignant arrhythmic events [26]. These results agree with numerous other reports in the literature regarding an idiopathic elevation of the J point and ST segment in the inferior leads with an increased tendency to ventricular arrhythmias [27,28,29,30]. An ECG showing a significant ST-segment elevation in a patient with Brugada syndrome is shown in Figure 4.

Also, antiarrhythmic drugs used to unleash the Brugada pattern may produce ST-segment elevation in the inferior leads, as shown by Nakamura et al., and this may mimic inferior ischemia [31].

Moreover, Potet et al. showed a SCN5A missense mutation producing a coved ST-segment elevation with a prominent J wave in leads II, III, and aVF [32].

A ST-segment elevation due to early repolarization (early repolarization pattern), which has long been considered benign [33], may be present in patients with Brugada pattern and has prognostic relevance.

Its electrocardiographic definition has been subject to significant variability among authors; it is important to recall that the true early repolarization pattern is defined by the presence of a notch or slur in the terminal QRS complex (J wave), along with ST-segment alterations [33,34]. It has been described that the presence of high-voltage J waves with horizontal or descending ST segments is more frequently observed in patients with idiopathic VF, while an ascending ST segment is more frequently observed in athletes and young adults and would have a benign significance [35,36]. Also, the dynamism and distribution of J waves and the presence of short-coupled premature ventricular beats are predisposing factors of ventricular arrhythmias [36].

Some authors have considered the hypothesis that BrS and early repolarization represent different phenotypes of the same genetic pathology [34]. However, this hypothesis remains unproven.

Serkozy et al. observed that, in a population with a spontaneous Brugada type 1 pattern, approximately 11% of subjects spontaneously exhibited an early repolarization pattern in the inferior and lateral leads. Moreover, patients with such characteristics appear to be at a higher risk of arrhythmic events. Additionally, subjects with early repolarization in the inferolateral leads seem more likely to unmask the Brugada pattern after the administration of Class 1 antiarrhythmics [37]. Conversely, other authors have not reported a worsening of outcomes in this population [38].

Kawata et al. analyzed a population with previous VF, demonstrating that the altered repolarization pattern can be consistently present, intermittent, or never documented. In this patient group, the presence of early repolarization in the inferolateral leads, either persistent or intermittent, was correlated with an increased risk of arrhythmic event recurrence (hazard ratio 4.88, *p* = 0.0004; and hazard ratio 2.50, *p* = 0.043, respectively) [39].

Numerous variables and confounding factors complicate the association between early repolarization, the Brugada pattern, and increased arrhythmic risk. These include the definition of early repolarization itself and the heterogeneity of populations studied, since the prognosis of the two conditions varies significantly based on sex, ethnicity, age, and clinical context. Moreover, confounding factors such as autonomic tone, body temperature, or circadian variation can significantly alter the ECG appearance of both conditions [40,41,42].

Further clinical studies are necessary to better investigate the association between these two electrocardiographic phenomena.

## 6. QRS Fragmentation in Patients with Brugada Syndrome

Fragmented QRS (fQRS) refers to the presence of additional notches or deflections within the QRS complex on surface ECG, often seen as an extra R wave (R’), notching in the nadir of the S wave, or more than one R’ in two contiguous leads corresponding to a specific myocardial region [43,44]. fQRS is known to be a marker of conduction delay inside the myocardium [43], associated with the presence of scarred areas, which determine a slowing down of the propagation of the electrical impulse within the muscle [44]. This finding has been independently associated with an increased risk of sudden cardiac death, both in chronic coronary syndromes and non-ischemic cardiomyopathies [45].

Patients with BrS may exhibit a variable degree of fragmentation of the QRS complex, especially in the right precordial leads, and this seems to be linked with a higher risk of ventricular tachyarrhythmias and sudden cardiac death [46]. The electrogenesis depends on the conduction abnormalities of the epicardium of the right ventricular outflow tract (RVOT), and it may also be linked to the fibrosis of that region of the ventricle [47].

**Figure 5:**
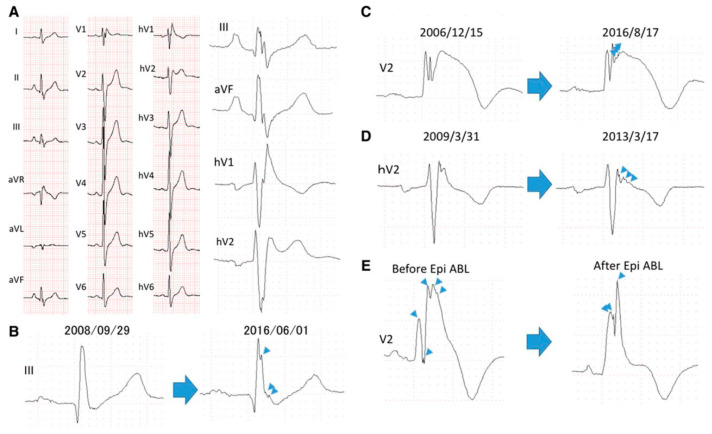
(**A**). ECG recorded in a 61-year-old male who experienced syncope at night. VF was detected 57 months after ICD implantation. hV1–hV6 are placed one intercostal space higher than the regular lead positions (V1–V6). Multiple R waves existed in leads II, III, aVF, V1, hV1, and hV2, and fragmented QRS were present in leads reflecting alteration in the inferior and RVOT regions. (**B**–**D**). Progressive increments of QRS fragmentation (arrowheads indicate new QRS spikes that appeared during follow-up). (**E**). Effect of epicardial ablation of the right ventricle: Before ablation, five spikes (arrowheads) were observed within QRS complex in lead V2. After ablation, only three spikes (arrowheads) can be seen, with a reduction in ST-segment elevation. Reproduced with permission from Morita et al. [48].

A recent study by Morita et al. showed that the fragmentation can involve the peripheral leads as well, in particular the inferior leads which explore the inferior wall of the left ventricle, carrying a higher risk of arrhythmic events. Moreover, the extension of the fQRS to multiple leads is associated with a worse prognosis: patients with fQRS had a shorter time to the onset of arrhythmic events (2.77%/year) than patients without fragmented QRS (0.46%/year) [48]. Since the electroanatomic substrate of BrS may progress over time [49], the extension of the fragmentation may also increase [48]. Therefore, it may be reasonable to associate the progression of the arrhythmic substrate, represented by an increase in QRS fragmentation, with an increase in arrhythmic risk, although this is not yet proven. It was also observed that the fQRS may be reduced in cases of improvement of the substrate, such as following epicardial ablation, as shown in Figure 5.

Similar considerations to those already made for the early repolarization pattern also apply to the fQRS. Indeed, confounding factors related to the definition of the fQRS itself, the heterogeneity of the population, and the patient’s condition can alter the results of the studies currently available in the literature.

## 7. Alterations of Atrioventricular Conduction

**Figure 6:**
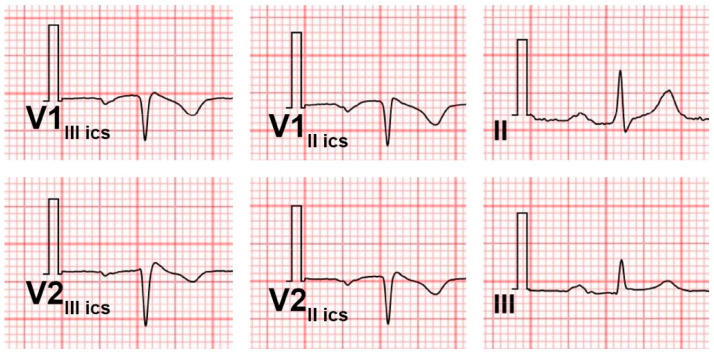
ECG from a 40-year-old patient with pathogenic mutation affecting the SCN5A gene, showing a first-degree atrioventricular block associated with a Brugada pattern. In the inferior leads, a slight notching of the P wave is also noted, which is prolonged, indicative of a possible intra-atrial conduction delay.

Patients with BrS may also present atrioventricular conduction impairments. The SCN5A mutation, primarily known for its association with BrS, has been associated with various degrees of conduction abnormalities on surface ECG, ranging from first-degree atrioventricular block (AVB), as shown in Figure 6, to complete AVB [50]. This mutation may impair sodium-channel function not only in ventricular myocardium but also in the cardiac conduction system, slowing conduction velocity [51].

Syncopal episodes in patients with BrS may be due to arrhythmic syncope, typically caused by aborted episodes of ventricular tachycardia or fibrillation, or the syncope may result from severe bradycardia, such as in complete AVB or sinus node dysfunction [52,53]. The latter can be hypothesized in cases of a prolonged HV interval on electrophysiological study and the presence of SCN5A pathogenic variants [50,51]. Clinically, tachyarrhythmic syncope benefits from the implantation of a subcutaneous defibrillator, which is not indicated in patients with syncope due to AVB, as it cannot provide pacing [54]. However, a modular system with a leadless pacemaker in wireless communication with a subcutaneous defibrillator may overcome this issue [55].

Furthermore, even the presence of first-degree AVB should be taken seriously, as it may progress to complete AVB [56], and it is a univariate predictor of arrhythmic events (hazard ratio 3.84; *p*  =  0.006), as shown by Migliore et al. [57].

Another important finding relates to the impact of SCN5A mutations on intra-atrial conduction. Studies have shown that these mutations can cause a slowing of electrical conduction within the atria, manifesting as a widened P wave on the ECG [58]. Abnormal atrial conduction causes an increased atrial vulnerability, becoming an electrophysiologic basis for an increased atrial fibrillation risk in patients with BrS [59]. This adds another layer of complexity to the long-term management of patients with BrS who develop supraventricular tachyarrhythmias.

Table 1 summarizes the electrocardiographic findings associated with an increased arrhythmic risk in patients with BrS, as discussed in detail throughout the text.

## 8. Conclusions

Brugada syndrome can become a diagnostic challenge when it occurs in association with electrocardiographic disturbances or with atypical presentations. While the coved-type ST-segment elevation in the right precordial leads remains the hallmark, this review highlights how conduction disturbances—such as RBBB or LBBB—can obscure the Brugada pattern. Moreover, the pattern may involve peripheral leads, with atypical presentations, and may be associated with alterations in the QRS and in atrioventricular and intra-atrial conduction. Recognizing these ECG findings is critical to avoid a misdiagnosis and for the correct management of the patient. Advanced techniques, including pharmacologic challenges and specific pacing maneuvers, can help unmask concealed patterns. Future studies are warranted to further clarify the prognostic implications of these atypical presentations and to optimize diagnostic strategies in affected patients.

## 9. Future Directions

Future research on Brugada syndrome should focus on improving the detection and characterization of atypical ECG presentations, especially in the presence of conduction disturbances like RBBB and LBBB that can mask the classic Brugada pattern. Advanced diagnostic techniques, such as novel pharmacologic challenges and specialized pacing maneuvers, warrant further exploration to reliably unmask concealed Brugada patterns. Additionally, longitudinal studies are needed to better understand the prognostic significance of peripheral lead abnormalities, QRS fragmentation, and atrioventricular conduction delays in this patient population. Genetic investigations, particularly into SCN5A mutations and their diverse electrophysiological impacts, could help refine risk stratification and prognostic implications. Ultimately, integrating these insights may optimize early diagnosis, improve risk prediction, and enhance clinical management strategies for patients with Brugada syndrome exhibiting atypical or overlapping conduction abnormalities.

## Figures and Tables

**Table 1:** A summary of the risk of arrhythmic events associated with electrocardiographic changes in patients with Brugada syndrome (see text for further details). HR = hazard ratio.

Associate Finding	Arrhythmic Risk	References
ST elevation in peripheral leads	Increased	[27,28,29,30]
Early repolarization	Increased (HR intermittent 2.5; persistent 4.88)	[39]
QRS fragmentation	Shorter time to arrhythmic events (2.77%/year vs. 0.46%/year)	[48]
AV Block	1st degree: predictor of arrhythmic events (HR 3.84)	[57]

## Abbreviations

The following abbreviations are used in this manuscript:

BrS	(Brugada Syndrome)
ECG	(Electrocardiogram)
RBBB	(Right Bundle Branch Block)
LBBB	(Left Bundle Branch Block)
RVOT	(Right Ventricular Outflow Tract)
AVB	(Atrioventricular Block)
